# Exogenous Nitric Oxide Suppresses in Vivo X-ray-Induced Targeted and Non-Targeted Effects in Zebrafish Embryos

**DOI:** 10.3390/ijms17081321

**Published:** 2016-08-12

**Authors:** E.Y. Kong, W.K. Yeung, T.K.Y. Chan, S.H. Cheng, K.N. Yu

**Affiliations:** 1 Department of Physics and Materials Science, City University of Hong Kong, Hong Kong, China; yikong3-c@my.cityu.edu.hk (E.Y.K.); wkyeung46-c@my.cityu.edu.hk (W.K.Y.); kayauchan4-c@my.cityu.edu.hk (T.K.Y.C.); 2 Department of Biomedical Sciences, City University of Hong Kong, Hong Kong, China; 3 State Key Laboratory in Marine Pollution, City University of Hong Kong, Hong Kong, China

**Keywords:** zebrafish embryos, nitric oxide, ionizing radiation, bystander effect

## Abstract

The present paper studied the X-ray-induced targeted effect in irradiated zebrafish embryos (*Danio rerio*), as well as a non-targeted effect in bystander naïve embryos partnered with irradiated embryos, and examined the influence of exogenous nitric oxide (NO) on these targeted and non-targeted effects. The exogenous NO was generated using an NO donor, *S*-nitroso-*N*-acetylpenicillamine (SNAP). The targeted and non-targeted effects, as well as the toxicity of the SNAP, were assessed using the number of apoptotic events in the zebrafish embryos at 24 h post fertilization (hpf) revealed through acridine orange (AO) staining. SNAP with concentrations of 20 and 100 µM were first confirmed to have no significant toxicity on zebrafish embryos. The targeted effect was mitigated in zebrafish embryos if they were pretreated with 100 µM SNAP prior to irradiation with an X-ray dose of 75 mGy but was not alleviated in zebrafish embryos if they were pretreated with 20 µM SNAP. On the other hand, the non-targeted effect was eliminated in the bystander naïve zebrafish embryos if they were pretreated with 20 or 100 µM SNAP prior to partnering with zebrafish embryos having been subjected to irradiation with an X-ray dose of 75 mGy. These findings revealed the importance of NO in the protection against damages induced by ionizing radiations or by radiation-induced bystander signals, and could have important impacts on development of advanced cancer treatment strategies.

## 1. Introduction

Nitric oxide (NO) is an important biological mediator in biological systems, and is involved in many different pathways such as immune response, neurotransmission and vasodilatation [1]. NO is endogenously generated from l-arginine by NO synthase (NOS) isoenzymes. NOS can be categorized into two functional classes, namely, the calcium-dependent constitutive NOS (cNOS) and the calcium-independent inducible NOS (iNOS) [2]. NO can diffuse through the cytoplasm and plasma membranes over distances of a few cell diameters [3].

NO is involved in both targeted effects as well as non-targeted bystander effects induced by ionizing radiations. Radiation-induced bystander effect (RIBE) in cells/organisms refer to the phenomenon that unirradiated cells/organisms respond as if they have been irradiated after having been partnered with the irradiated cells/organisms or after having been introduced into the medium previously conditioning the irradiated cells/organisms. Ionizing radiations can activate the ataxia telangiectasia mutated (ATM)-NF-κB signaling pathway [4]. NF-κB enters the nucleus and acts as a transcription factor for cyclooxygense-2 (COX-2) and iNOS genes. After its induction, iNOS can produce sustained high concentrations of NO. On the other hand, ionizing radiations can transiently stimulate the activity of cNOS [5]. Secreted or membrane-associated forms of cytokines, such as TNFα, can also activate IκB kinase (IKK)-mediated phosphorylation of IκB, which releases NF-κB that enters the nucleus. TNFα also activates MAPK pathways that, via AP-1 transcription factor, additionally upregulate expression of COX-2 [6] and iNOS. There was also evidence that NO could induce expression of COX-2 in cells [7,8]. Activation of COX-2 provided a continuous supply of reactive radicals and cytokines for the propagation of bystander signals [9]. NO has some intriguing properties in that it can lead to opposite biological functions, e.g., NO can be both pro-apoptotic and anti-apoptotic [10,11].

In a previous in vivo study [12], we examined the influence of NO on the bystander effect between embryos of the zebrafish, *Danio rerio*, irradiated with high-dose X-rays and naive unirradiated embryos. We demonstrated that the RIBE between partnered irradiated and naïve unirradiated zebrafish embryos was suppressed through the addition of the NO scavenger 2-(4-carboxyphenyl)-4,4,5,5-tetramethylimidazoline-1-oxyl-3-oxide (cPTIO) into the medium [12]. As RIBE was also induced in zebrafish naïve embryos introduced into the irradiated embryo conditioned medium (IECM) alone, i.e., without directly partnering with the irradiated embryos, and in view of the short life of NO in the IECM, NO should have been involved in the generation of bystander signals in irradiated embryos, or in generation of bystander response (in terms of apoptosis) in naïve unirradiated embryos upon receiving bystander signals, or both [12]. In relation to this, previous in vitro studies by other groups also demonstrated that pretreatment with c-PTIO could eliminate the RIBE [13,14,15].

In a subsequent in vivo study, Choi et al. [16] further investigated the radioadaptive response (RAR) induced in zebrafish embryos by 3.4 MeV protons at 5 h post fertilization (hpf) against a challenging exposure of 2 Gy of X-ray irradiation at 10 hpf. The RAR (in terms of mitigation of apoptosis) was suppressed by adding cPTIO to the medium at 5 h after the priming exposure, when de novo synthesis of factors required by RAR should have been completed, which suggested that NO was required for the repair machineries against apoptosis in the bystander cells. The results from these two in vivo studies showed that NO could be both pro-apoptotic and anti-apoptotic in bystander zebrafish embryos. As such, it would be pertinent to examine the influence of exogenous NO on RIBE in zebrafish embryos.

In the present work, the influence of exogenous NO on X-ray-induced targeted effects and non-targeted bystander effects in zebrafish embryos were studied. The exogenous NO was generated using a NO donor, *S*-nitroso-*N*-acetylpenicillamine (SNAP). SNAP could spontaneously release NO within 15 min upon light stimulation, and 100 μM of SNAP could generate ~27 μM of NO [17]. Zebrafish has become a popular vertebrate model in genetic, pharmacological and behavioral studies. In relation, zebrafish embryos have also been widely employed for examining biological effects of ionizing radiations [18,19,20,21,22,23]. The most important advantage of using zebrafish as an animal model in studying the biological effects is that zebrafish share considerable genetic sequence similarity with humans [24,25]. Other advantages include high fecundity, low maintenance cost, transparent embryos and rapid development.

## 2. Results

**Figure 1:**
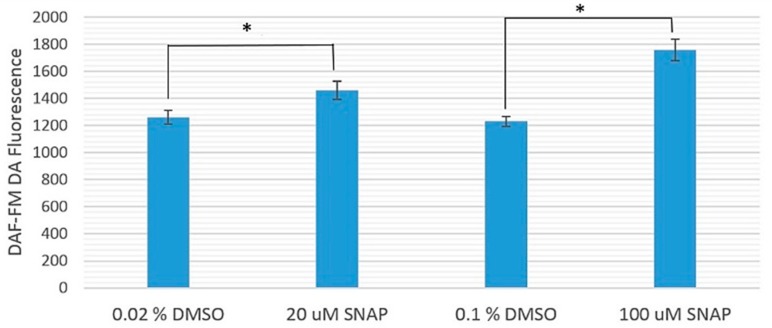
Comparison between the diaminofluorophore 4-amino-5-methylamino-2′-7′-difluorofluorescein diacetate (DAF-FM DA) fluorescence intensities (in arbitrary units; larger values corresponding to brighter fluorescence) in zebrafish embryos treated with 20 or 100 μM S-nitroso-*N*-acetylpenicillamine (SNAP) and their corresponding experimental controls treated with 0.02% or 0.1% dimethyl sulfoxide (DMSO), respectively. All experiments were carried out in triplicate on different days, and each set consisted of 15 examined zebrafish embryos. The data were shown as mean DAF-FM DA fluorescence intensities ± standard error of the mean (SEM). Cases with *p* < 0.05 are asterisked.

Figure 1 shows the comparison between the fluorescence intensities from diaminofluorophore 4-amino-5-methylamino-2′-7′-difluorofluorescein diacetate (DAF-FM DA), which surrogated the NO levels, in zebrafish embryos treated with 20 or 100 μM SNAP and their corresponding experimental controls treated with 0.02% or 0.1% dimethyl sulfoxide (DMSO), respectively. The average DAF-FM DA fluorescence intensities from zebrafish embryos treated with 20 and 100 μM SNAP were significantly larger than their corresponding experimental controls (treated only with 0.02% and 0.1% DMSO, respectively), with *p*-values smaller than 0.039 and 0.0061, respectively. In other words, the NO levels in SNAP-treated zebrafish embryos were indeed higher than those in the experimental controls.

**Figure 2:**
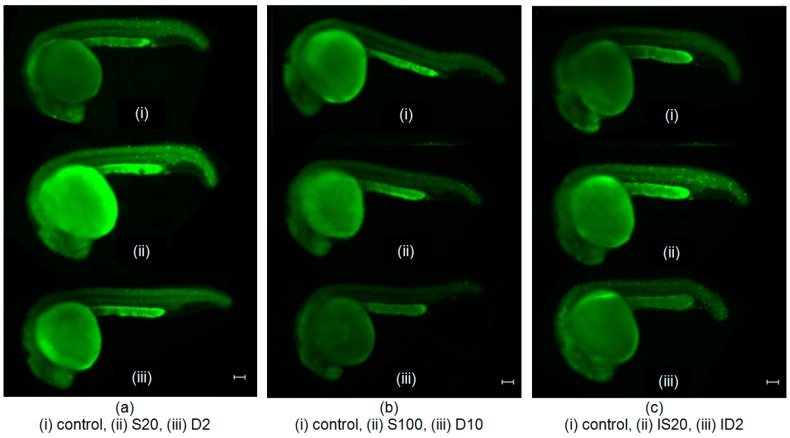

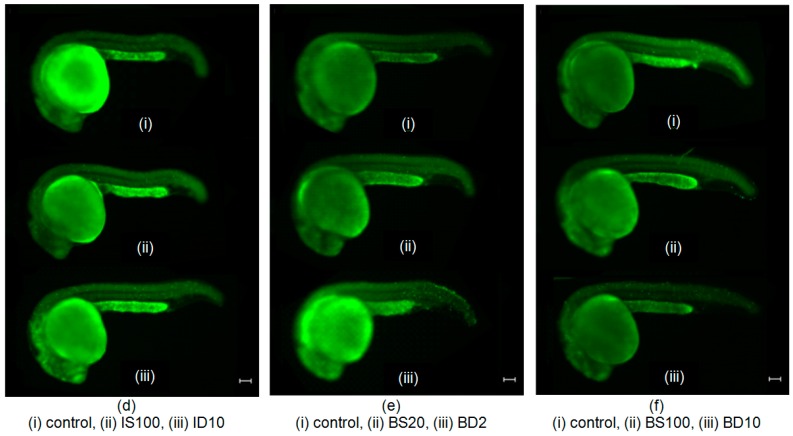
Representative images of acridine orange (AO)-stained embryos in different groups. (**a**) S20 group treated with 20 μM SNAP, and its experimental control D2 group; (**b**) S100 group treated with 100 μM SNAP, and its experimental control D10 group; (**c**) IS20 irradiated group treated with 20 μM SNAP, and its experimental control ID2 group; (**d**) IS100 irradiated group treated with 100 μM SNAP, and its experimental control ID10 group; (**e**) BS20 bystander group treated with 20 μM SNAP, and its experimental control BD2 group; (**f**) BS100 bystander group treated with 100 μM SNAP, and its experimental control BD10 group. (**a**–**f**) all corresponding control groups also shown. Images were captured using a florescent microscope with 40× magnification. Scale bar: 100 µm.

Figure 2 shows representative images of acridine orange (AO)-stained embryos in different groups: (a) S20 group treated with 20 μM SNAP, and its experimental control D2 group (treated with 0.02% DMSO); (b) S100 group treated with 100 μM SNAP, and its experimental control D10 group (treated with 0.1% DMSO); (c) IS20 irradiated group treated with 20 μM SNAP, and its experimental control ID2 group (irradiated and treated with 0.02% DMSO); (d) IS100 irradiated group treated with 100 μM SNAP, and its experimental control ID10 group (irradiated and treated with 0.1% DMSO); (e) BS20 bystander group treated with 20 μM SNAP, and its experimental control BD2 group (treated with 0.02% DMSO); and (f) BS100 bystander group treated with 100 μM SNAP, and its experimental control BD10 group (treated with 0.1% DMSO); and all the corresponding control groups.

### 2.1. Cytotoxicity of 20 and 100 μM SNAP on Zebrafish Embryos

The cytotoxic effects of SNAP with concentrations of 20 and 100 μM were examined. For each concentration of 20 and 100 μM of SNAP, three sets of experiments were performed separately. The differences among all groups of embryos were assessed using analysis of variance (ANOVA) and the results obtained for 20 and 100 μM of SNAP showed that the *p*-values were >0.05 (Table 1 and Table 2, Figure 2a,b), which indicated that SNAP with concentrations of 20 and 100 μM did not have significant effects on zebrafish embryos.

### 2.2. Effects of SNAP on Zebrafish Embryos Irradiated with 75 mGy X-ray

To examine whether NO could alleviate the X-ray induced targeted effect, we first treated the zebrafish embryos with SNAP at 3 hpf for 2 h. The embryos were then transferred into fresh E3 medium and irradiated with X-ray. Table 3 and Figure 2c showed that when the embryos were treated with 20 μM of SNAP before being irradiated with X-rays, the number of apoptotic events was smaller than that obtained in the experimental control but there was no significant difference between these two groups. As such, 20 μM of SNAP could not mitigate the damage in terms of apoptosis when the embryos were exposed to 75 mGy X-ray irradiation. In contrast, Table 4 and Figure 2d showed that when the embryos were treated with 100 μM of SNAP before being irradiated with X-rays, the number of apoptotic events was significantly smaller than that obtained in the experimental control. This indicated that 100 μM of SNAP could alleviate the X-ray induced damages in the zebrafish embryos.

### 2.3. Effects of SNAP on Radiation-Induced Bystander Effect in Zebrafish Embryos

To examine whether NO could protect the unirradiated naïve zebrafish embryos having partnered with irradiated embryos, we also pretreated the naïve zebrafish embryos with SNAP with concentrations of 20 and 100 μM at 3 hpf for 2 h. At 5 hpf, the pretreated naïve embryos were transferred into fresh E3 medium, which were then partnered with the irradiated embryos for 19 h. Table 5 and Table 6, and Figure 2e,f showed that the numbers of apoptotic events on the embryos treated with 20 and 100 μM of SNAP were significantly smaller than those for the experimental control groups for all sets of experiments. In other words, SNAP with a concentration of 20 or 100 μM effectively suppressed the bystander effects in the naïve embryos partnered with the irradiated embryos.

## 3. Discussion

The present work studied the influence of NO on X-ray-induced targeted effects in irradiated zebrafish embryos and non-targeted effect in bystander naïve embryos partnered with irradiated embryos. These effects were assessed using the number of apoptotic events in the zebrafish embryos at 24 hpf revealed through AO staining, which was a common adopted biological endpoint for studying radiation-induced effects in zebrafish embryos [20].

The present work showed that zebrafish embryos did not exhibit deleterious effects in terms of apoptotic events when treated by 20 and 100 μM of SNAP. The targeted effect was alleviated if the zebrafish embryos were pretreated with 100 µM (but not with 20 µM) SNAP prior to irradiation with an X-ray dose of 75 mGy. The results agreed with previous results from other groups [26,27,28]. Liebmann et al. [26] demonstrated that pretreatment with the NO-releasing agent, (C_2_H_5_)_2_N[N(O)NO^–^]Na^+^ (DEA/NO), enhanced the survival of mice having been subjected to whole body irradiation. Tokumizu et al. reported that treatment of RAW264.7 cells with NO radical donors reduced the micronuclei frequency induced by γ-irradiation [27]. Suschek et al. showed that NO could protect endothelial cells against UVA-induced apoptosis [28]. These findings confirmed that exogenous NO could act as a protector against radiation-induced targeted effects.

The present work also showed that the non-targeted effect in terms of apoptotic events was eradicated if the bystander naïve zebrafish embryos were pretreated with 20 or 100 µM SNAP for 2 h prior to their partnering with zebrafish embryos having already been irradiated with an X-ray dose of 75 mGy. Interestingly, previous research suggested that scavenging NO with cPTIO could suppress RIBE in the bystander zebrafish embryos [12]. Taken together, both scavenging NO from bystander embryos and adding exogenous NO to bystander embryos were anti-apoptotic.

These findings were intriguing in that the biological effect (in terms of apoptosis) changed in the same direction (suppression of apoptosis) regardless of removal of NO from or addition of NO to the zebrafish embryos. The toxicity of NO could be attributed to peroxynitrite (ONOO^−^) formed by the combination of NO and O_2_^−^, which was an oxidizing free radical that could cause DNA fragmentation and lipid peroxidation, protein nitration and cell death [29,30]. As such, suppression of apoptosis would be expected through scavenging NO from the targeted embryos or non-targeted bystander embryos. On the other hand, mechanisms were proposed for suppression of apoptosis through adding exogenous NO. For example, NO could be involved in the activation of Hdm2, inhibition of p53 activation and/or inactivation of the p53 protein [31,32]*.* Furthermore, mechanisms were also proposed where NO was involved in repairing DNA damage. Xu et al. showed that exposure of cells to NO resulted in a 4–5-fold increase in the expression of the DNA-dependent protein-kinase catalytic subunit (DNA-PKcs), which was one of the important enzymes involved in repairing DNA double strand breaks [33]. Matsumoto et al. reported that accumulation of the heat-shock protein HSP72 and wild-type TP53 in NO recipient cells co-cultivated with heat-shocked NO donor cells were induced through an intercellular signal transduction pathway initiated by NO [34,35]. Gansauge et al. [36] reported that endogenous production of NO caused G1-phase arrest in human carcinoma cell lines. During G1-phase arrest induced by NO, the accumulated TP53 and HSP72 might induce DNA repair machinery that repaired DNA damages and protein repair machinery that repaired denatured proteins, respectively [34,35].

The toxicity of NO due to the formation of ONOO^−^, its capability to suppress apoptosis through activation of Hdm2, inhibition of p53 activation and/or inactivation of the p53 protein, as well as its involvement in repairing of DNA damage through expression of DNA-PKcs, accumulation HSP72 and wild-type TP53 and/or G1-phase arrest lead to opposite biological effects (in terms of apoptosis). This might explain why the biological effects (in terms of apoptosis) changed in the same direction regardless of removal of NO from or addition of NO to the zebrafish embryos. For example, although it would be expected that increasing the NO level in embryos would lead to an increase in the ONOO^−^ toxicity and thus an increased number of apoptotic events, the increased NO level would also suppress apoptosis and facilitate DNA repair through the mechanisms described above, so the resultant final outcome could still be a decreased number of apoptotic events.

These results could have important impacts on development of advanced cancer treatment strategies in which NO could play various critical roles. Some studies showed that NO could be a potent tumor radiosensitizer [37,38], but reduction of NO levels could also lead to radiosensitization [39]. More studies would be needed before final conclusions could be made.

## 4. Materials and Methods

### 4.1. Zebrafish Maintenance

The animal studies in Hong Kong were approved by the Department of Health, Government of the Hong Kong Special Administrative Region, with the ref. No. Ref: (13-7) in DH/HA&P/8/2/5 Pt.1, and were performed in accordance with the guidelines.

To provide the embryos required for the experiments in the present work, about 30 adult zebrafish were kept in a 45 L fish tank under a 14/10-light/dark cycle. The water temperature was maintained at 28.5 °C using a thermostat. The zebrafish were fed with dry fish food four times and brine shrimp once daily. To collect the embryos, a specially designed plastic collector was used [19]. To ensure synchronization of the stages of the collected embryos, the collector was left in the fish tank for only 15 min from the start of light-induced spawning. The collected embryos were transferred to a Petri dish with E3 medium (5 mM NaCl, 0.33 mM MgSO_4_, 0.33 mM CaCl_2_, 0.17 mM KCl, and 0.1% methylene blue) and then manually dechorionated using a pair of forceps (Dumont, Hatfield, PA, USA) under a stereomicroscope. The dechorionated embryos were then returned into the incubator maintained at 28.5 °C for further development until 3 hpf.

### 4.2. Treatment with SNAP

A 0.1 M SNAP stock solution was prepared by dissolving SNAP (Thermo Fisher Scientific, cat No.: N7892, Eugene, OR, USA) in DMSO and was stored at −20 °C. In the present experiments, 20 and 100 μM SNAP solutions were employed, which were freshly prepared by dissolving the SNAP stock solution in E3 solution each time before the experiments. For each studied group of zebrafish embryos to be treated by SNAP, a total of 15 dechorionated embryos at 3 hpf were accommodated in a 35 mm Petri dish with 2 mL solution (20 or 100 μM SNAP) at 3 hpf for 2 h. For the corresponding experimental control embryos, the 20 or 100 μM SNAP were replaced with 0.02% or 0.1% DMSO, respectively. For studies on the cytotoxicity or the targeted effects, each studied group consisted of no more than 12 embryos transferred from the Petri dish into fresh E3 medium. For the studies on the non-targeted effects (RIBE), each studied group consisted of 10 embryos transferred from the Petri dish into fresh E3 medium to match the same number (10) of partnered irradiated embryos. In order to confirm that the NO levels in SNAP-treated zebrafish embryos were indeed higher than those in the experimental controls, we performed an extra experiment to label the zebrafish embryos with DAF-FM DA (Thermo Fisher Scientific, lot No.: 1351904) to reveal their NO levels. When the zebrafish embryos developed to 3 hpf, SNAP or DMSO stock solutions loaded with 5 μM DAF-FM DA were added into the E3 medium. After incubation for 2 h in dark at 28.5 °C, the embryos were rinsed with fresh E3 medium before in vivo visualization. The average DAF-FM DA fluorescence intensities from zebrafish embryos were analyzed using the NIS-Elements Basic Research software (version 4.40.00, Nikon Instruments Inc., Melville, NY, USA).

### 4.3. X-ray Irradiation

After SNAP treatment for 2 h, the zebrafish embryos were transferred into a fresh E3 medium and were then X-ray irradiated using an X-ray generator, namely, X-RAD 320 irradiator (Precision X-ray Inc., North Branford, CT, USA), with voltage and current set at 150 kV and 2 mA, respectively, while the source–surface distance was set at 70 cm. In the present experiments, an X-ray filter made of 2 mm thick aluminum (Al) was also employed to harden the X-ray [40]. With these operation parameters, the dose rate was ~46 mGy/min and the dose received by the embryos was 75 mGy. For a reference, under the same irradiation parameters, Kong et al. [40] revealed that X-ray doses larger than 50 mGy induced detrimental effects in zebrafish embryos. The dose rates were monitored using the PTW UNIDOSE Universal Dosemeter (SN006861, PTW, Freiburg, Germany). Throughout the experiments, the embryos were kept at the room temperature. After irradiation, the embryos were incubated at 28.5 °C until 24 hpf for further analyses.

### 4.4. Cytotoxicity of SNAP

**Figure 3:**
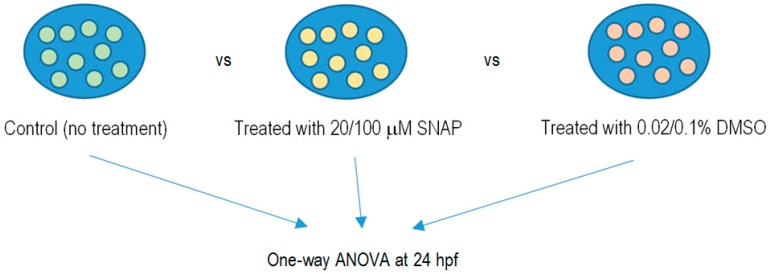
Comparison among different groups of embryos to test the cytotoxicity of SNAP.

To examine whether SNAP with concentrations of 20 and 100 μM were harmful to the zebrafish embryos, embryos at 3 hpf were incubated with SNAP with concentrations of 20 or 100 μM in 35 mm Petri dish for 2 h. In the experimental control experiments, the SNAP treatments were replaced with 0.02% and 0.1% DMSO, respectively. Furthermore, another control group without any treatment was also set up to compare with the group of SNAP-treated embryos. The experimental protocol is illustrated in Figure 3.

### 4.5. X-ray-Induced Targeted Effects and Non-Targeted Bystander Effects

**Figure 4:**
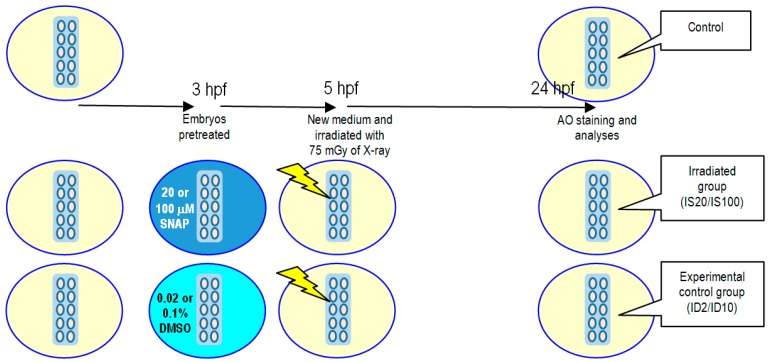
Schematic diagram showing the protocols for studying the influence of SNAP on X-ray-induced targeted effects. Embryos were pretreated with 20 or 100 μM SNAP to form the irradiated groups IS20 or IS100, respectively, or pretreated with 0.02% or 0.1% DMSO for 2 h at 3 hpf to form the experimental control groups ID2 or ID10, respectively. After pretreatment, the irradiated groups and the experimental control groups were transferred to new E3 media and were then irradiated with 75 mGy of X-ray. The embryos were then incubated at 28.5 °C until 24 hpf for AO staining and analyses.

For studies on X-ray-induced targeted effects, the experimental protocol is illustrated in Figure 4. Embryos were pretreated with 20 or 100 μM SNAP to form the irradiated groups IS20 or IS100, respectively, or pretreated with 0.02% or 0.1% DMSO for 2 h at 3 hpf to form the experimental control groups ID2 or ID10, respectively. After pretreatment, the irradiated groups and the experimental control groups were transferred to new E3 media and were then irradiated with 75 mGy of X-ray. The embryos were then incubated at 28.5 °C until 24 hpf for vital dye AO staining and analyses.

**Figure 5:**
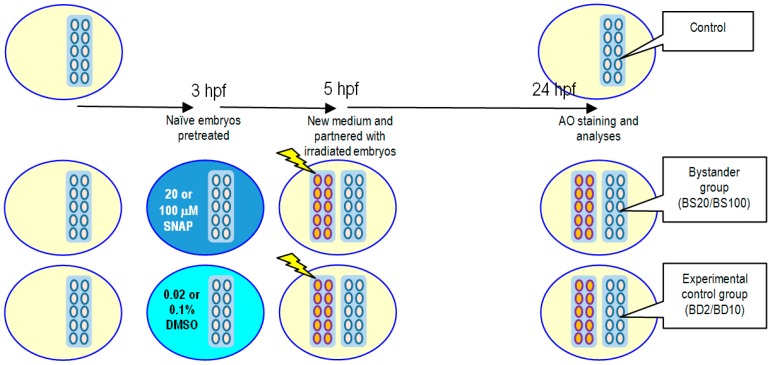
Schematic diagram showing the protocols for studying the influence of SNAP on X-ray-induced non-targeted bystander effects. Naïve embryos were pretreated with 20 or 100 μM SNAP to form the bystander groups BS20 or BS100, respectively, or pretreated with 0.02% or 0.1% DMSO for 2 h at 3 hpf to form the experimental control groups BD2 or BD10, respectively. After pretreatment, the bystander groups and the experimental control groups were transferred to new E3 media and were then partnered for 19 h with embryos already irradiated with 75 mGy of X-ray. The embryos were then incubated at 28.5 °C until 24 hpf for AO staining and analyses.

For studies on X-ray-induced non-targeted bystander effects, the experimental protocol is illustrated in Figure 5. Naïve embryos were pretreated with 20 or 100 μM SNAP to form the bystander groups BS20 or BS100, respectively, or pretreated with 0.02% or 0.1% DMSO for 2 h at 3 hpf to form the experimental control groups BD2 or BD10, respectively. After pretreatment, the bystander groups and the experimental control groups were transferred to new E3 media and were then partnered for 19 h with embryos already irradiated with 75 mGy of X-ray. The embryos were then incubated at 28.5 °C until 24 hpf for vital dye AO staining and analyses.

### 4.6. Vital Dye Acridine Orange (AO) Staining

Apoptosis in the 24 hpf embryos was employed as the biological endpoint in the present work. The AO dye (Sigma, St. Louis, MO, USA) with a concentration of 5 μg/mL was used to quantify apoptotic cells, which had been commonly employed to quantify the level of apoptosis in zebrafish embryos [41,42,43]. Zebrafish embryos at 24 hpf were transferred into each of the 24 wells containing the AO dye and were kept at 28.5 °C for 45 min. During staining, the embryos were kept in the dark in order to minimize fading of the AO color. The embryos were then washed twice using deionized water thoroughly to remove excessive AO. After anaesthetizing the embryos by 0.0016 M tricaine (Sigma, St. Louis, MO, USA), three images with focuses on different sections of each anaesthetized embryo were captured using SpotBasic (SPOT 4.7, Diagnostic Instruments Inc., Sterling Heights, MI, USA) with a magnification of 40× under a fluorescent microscope. The apoptotic events in each embryo were counted with the help of a computer program.

### 4.7. Data Analysis

Under each experimental condition, all experiments were carried out in triplicate on different days. The data were shown as the average numbers of apoptotic counts ± standard error of the mean (SEM). One-way analysis of variance (ANOVA) was used to check the cytotoxicity of different concentrations of SNAP. As regards the X-ray-induced targeted effects and non-targeted bystander effects, *t*-tests were used to check the differences between groups, where *p*-values < 0.05 were considered to correspond to significant differences between the compared groups.

## 5. Conclusions

The present paper studied X-ray-induced targeted and non-targeted effects in zebrafish embryos and examined the influence of exogenous NO generated using SNAP on these effects, with the number of apoptotic events in the embryos at 24 hpf revealed through AO staining as the biological endpoint. The targeted effect was mitigated if the embryos were pretreated with 100 µM SNAP prior to irradiation with an X-ray dose of 75 mGy but was not alleviated if the embryos were pretreated with 20 µM SNAP. On the other hand, the non-targeted effect was eliminated in the bystander naïve embryos if they were pretreated with 20 or 100 µM SNAP prior to partnering with embryos having been subjected to irradiation with an X-ray dose of 75 mGy. These findings revealed the importance of NO in the protection against damages induced by ionizing radiations or by radiation-induced bystander signals.

## Figures and Tables

**Table 1:** Mean number of apoptotic events (±SEM) obtained in the control, S20 and D2 groups from 3 sets of experiments. *n*: numbers of embryos involved in the analyses. *^#^ p* values obtained using ANOVA.

Set	Control	S20	D2	*p* ^#^
1	92.5 ± 13.6 (*n* = 10)	86.1 ± 11.8 (*n* = 8)	126 ± 19 (*n* = 8)	0.164
2	83.4 ± 5.4 (*n* = 7)	95.2 ± 10.4 (*n* = 6)	89.4 ± 7.2 (*n* = 7)	0.566
3	173 ± 11 (*n* = 11)	156 ± 11 (*n* = 10)	173 ± 18 (*n* = 10)	0.619

**Table 2:** Mean number of apoptotic events (±SEM) obtained in the control, S100 and D10 groups from 3 sets of experiments. *n*: numbers of embryos involved in the analyses. *^#^ p* values obtained using ANOVA.

Set	Control	S100	D10	*p* ^#^
1	96.0 ± 7.4 (*n* = 7)	108 ± 10 (*n* = 9)	110 ± 15 (*n* = 9)	0.689
2	65.5 ± 5.1 (*n* = 12)	64.5 ± 6.0 (*n* = 12)	62.7 ± 6.2 (*n* = 12)	0.941
3	87.5 ± 5.4 (*n* = 12)	84.5 ± 6.1 (*n* = 11)	86.6 ± 5.0 (*n* = 10)	0.922

**Table 3:** Mean number of apoptotic events (±SEM) obtained in the control, IS20 and ID2 groups from 3 sets of experiments. *n*: numbers of embryos involved in the analyses. *^#^ p* values obtained by comparing the IS20 and ID2 groups using two-tailed *t*-test.

Set	Control	IS20	ID2	*p* ^#^
1	87.9 ± 6.4 (*n* = 7)	186 ± 13 (*n* = 9)	202 ± 19 (*n* = 9)	0.517
2	108 ± 5 (*n* = 10)	196 ± 12 (*n* = 11)	198 ± 14 (*n* = 11)	0.898
3	83.4 ± 5.4 (*n* = 7)	136 ± 13 (*n* = 8)	157 ± 11 (*n* = 7)	0.236

**Table 4:** Mean number of apoptotic events (±SEM) obtained in the control, IS100 and ID10 groups from 3 sets of experiments. *n*: numbers of embryos involved in the analyses. *^#^* *p* values obtained by comparing the IS100 and ID10 groups using one-tailed *t*-test (cases with *p* < 0.05 are asterisked).

Set	Control	IS100	ID10	*p* ^#^
1	74.3 ± 4.3 (*n* = 10)	157 ± 28 (*n* = 10)	250 ± 37 (*n* = 9)	4.65 × 10^−3^ *
2	108 ± 5 (*n* = 10)	177 ± 8 (*n* = 10)	210 ± 10 (*n* = 11)	9.69 × 10^−3^ *
3	110 ± 9 (*n* = 7)	131 ± 9 (*n* = 10)	204 ± 13 (*n* = 10)	1.99 × 10^−4^ *

**Table 5:** Mean number of apoptotic events (±SEM) obtained in the control, BS20 and BD2 groups from 3 sets of experiments. *n*: numbers of embryos involved in the analyses. *^#^* *p* values obtained by comparing the BS20 and BD2 groups using the one-tailed *t*-test (cases with *p* < 0.05 are asterisked).

Set	Control	BS20	BD2	*p* ^#^
1	82.7 ± 10.4 (*n* = 10)	72.4 ± 7.2 (*n* = 9)	154 ± 15 (*n* = 9)	1.65 × 10^−4^ *
2	102 ± 11 (*n* = 10)	91.4 ± 7.1 (*n* = 8)	137 ± 12 (*n* = 9)	2.78 × 10^−3^ *
3	63.0 ± 2.9 (*n* = 9)	67.3 ± 4.3 (*n* = 9)	95.3 ± 7.6 (*n* = 8)	3.46 × 10^−3^ *

**Table 6:** Mean number of apoptotic events (±SEM) obtained in the control, BS100 and BD10 groups from 3 sets of experiments. *n*: numbers of embryos involved in the analyses. *^#^* *p* values obtained by comparing the BS100 and BD10 groups using one-tailed *t*-test (cases with *p* < 0.05 are asterisked).

Set	Control	BS100	BD10	*p* ^#^
1	129 ± 15 (*n* = 10)	113 ± 12 (*n* = 9)	239 ± 21 (*n* = 9)	7.14 × 10^−5^ *
2	103 ± 14 (*n* = 8)	104 ± 14 (*n* = 9)	156 ± 15 (*n* = 9)	9.77 × 10^−3^ *
3	96.0 ± 7.4 (*n* = 7)	87.6 ± 6.8 (*n* = 8)	129 ± 15 (*n* = 8)	1.67 × 10^−2^ *
